# Generalization of Conditioned Fear and Obsessive-Compulsive Traits

**Published:** 2013

**Authors:** Antonia N Kaczkurkin, Shmuel Lissek

**Affiliations:** Department of Psychology, University of Minnesota, USA

**Keywords:** Obsessive-compulsive disorder, Fear conditioning, Generalization, Fear-potentiated startle

## Abstract

Generalization of conditioned fear refers to the transfer of the conditioned fear response to stimuli that resemble the original conditioned stimulus. Overgeneralization of conditioned fear has been associated with panic disorder and generalized anxiety disorder and may be relevant to obsessive-compulsive (OC) symptoms as well. This study represents the first attempt to determine the degree to which individuals with high versus low OC traits over generalize conditioned fear. We hypothesized that the high OC individuals, particularly those characterized by overestimation of threat, would show overgeneralization of conditioned fear compared to controls as measured by behavioral and psychophysiological (fear-potentiated startle) measures. The results of this study show an interaction between the high and low Threat Estimation groups as measured by the Obsessive Beliefs Questionnaire, which suggests that those who have a tendency to overestimate threat show overgeneralization of conditioned fear. This finding suggests that the relation between OC symptoms and overgeneralization of conditioned fear may be specific to the high threat estimation component of OC symptoms.

## Introduction

### Generalization of conditioned fear and obsessive-compulsive traits

Fear-conditioning refers to emotional learning to a neutral stimulus (conditioned stimulus or CS) after it is paired with an unconditioned aversive stimulus (US), leading the neutral stimulus to elicit anxiety associated with the anticipation of the aversive event (conditioned response or CR). Classical fear-conditioning accounts of pathological anxiety have focused on abnormalities in the acquisition (overly strong acquisition of fear responses), inhibition (failure to inhibit fear responses), extinction (resistance to extinguish fear responses), avoidance (actively avoiding stimuli that would lead to fear responses), or overgeneralization of conditioned fear [1]. In particular, the overgeneralization of conditioned fear has been suggested to be an important feature of pathological anxiety. The generalization of conditioned fear refers to the transfer of the conditioned fear response to stimuli that resemble the original conditioned stimulus [2]. The goal of the current study is to investigate the generalization of fear conditioning in individuals with obsessive-compulsive traits as measured by the Obsessive-Compulsive Inventory-Revised [3].

Overgeneralization of conditioned fear has been associated with panic disorder [4] and generalized anxiety disorder [5] and may be relevant to other anxiety disorders as well, such as obsessive-compulsive disorder (OCD). OCD is a chronic and debilitating disorder characterized by intrusive thoughts and repetitive acts to reduce anxiety [6]. Anecdotal evidence for overgeneralization is apparent in descriptions of OCD symptoms. An individual with a fear of contamination from a certain object may then generalize that fear to other objects or people that resemble the original object. For example, an OCD patient who encounters a particularly unsanitary public restroom may develop a fear of contamination from using not only that one restroom but all public restrooms. The threat posed by the one restroom has been generalized to all restrooms despite safety cues in the environment (e.g., apparent cleanliness) that should inhibit the fear response to sanitary public restrooms.

Additionally, OCD has been linked to a tendency to overestimate threat [7,8] and the Obsessive-Beliefs Questionnaire (OBQ-44) recognizes excessive threat estimation as one domain of symptoms related to OCD [9]. Overestimation of threat refers to beliefs that one’s environment is unsafe, despite evidence to the contrary. In the context of generalization, those high on threat estimation should be over-reactive to stimuli resembling the danger cue even though the dissimilar part of the stimulus is actually a sign of safety. As such, a tendency toward overestimating threats may be an important precursor to conditioned generalization and may predict overgeneralization in OCD. Furthermore, neuroimaging research supports the association between OCD and the possible overgeneralization of fear responses. Patients with OCD show stronger amygdala involvement for both OCD-related images and general aversive images, which has been interpreted as evidence of “generalized emotional hyperresponsivity” to non-symptom specific stimuli [10].

Despite the intuitive relationship between fear generalization and OCD, there is currently no research that systematically investigates the generalization of fear in individuals with obsessive-compulsive traits using fear conditioning paradigms. The purpose of the current study is to determine the degree to which individuals with obsessive-compulsive (OC) traits generalize conditioned fear when compared to healthy participants. In this study, participants completed a generalized fear conditioning task based on discriminative fear-conditioning as described in Lissek et al. [4]. In discriminative conditioning, two conditioned stimuli are presented, one that is paired with the unconditioned stimulus (referred to as the CS+ or danger cue) and one that is not paired with the unconditioned stimulus (CS− or safety cue). Within-subject effects are measured in discrimination conditioning as the difference in fear-potentiated startle amplitudes to the danger versus safety cues. The fear-potentiated startle response is the reliable enhancement of the startle reflex when a person is in a state of fear [11–13] and has been shown to discriminate between healthy participants and those with different forms of psychopathology including internalizing and externalizing disorders, psychotic disorders, and personality disorders [14,15].

In addition to the presentation of CS+ and CS−, generalization stimuli forming a continuum of similarity between the CS+ and CS− are presented to test generalization effects. Specifically, this paradigm produces generalization gradients (slopes) where fear responses decrease as generalization stimuli become less similar to the conditioned danger cue [16]. Generalization gradients of conditioned fear have long been demonstrated in research using animals [17–19]. This work has been translated to human studies on generalization, which also demonstrates precipitous declines in fear as the generalization stimuli becomes less similar to the conditioned danger cue in healthy participants [16,20,21].

Furthermore, these generalization gradients have been shown to differentiate between healthy participants and both panic disorder and generalized anxiety disorder (GAD) patients. Healthy participants show steep quadratic declines in fear responses to the generalization stimuli while responses in panic and GAD patients are characterized by linear, more gradual declines in fear responses to the generalization stimuli [4,5]. In other words, as stimuli become less similar to the danger cue, healthy participants are able to differentiate between the danger cue and approximations of the danger cue and their fear responses quickly decline. However, panic and GAD patients are less able to emotionally differentiate between the danger cue and its approximations; thus, they continue to show high levels of fear to stimuli that resemble the danger cue, suggesting overgeneralization of conditioned fear. Furthermore, a generalization paradigm has been used to discriminate GAD patients from healthy controls in terms of activation of the ventral medial prefrontal cortex [22].

The current study hypothesizes that individuals with OC traits will show overgeneralization of conditioned fear compared to controls as measured by startle potentiation and self-report ratings. Specifically, healthy participants are predicted to show quadratic generalization gradients, suggesting normal, more precipitous declines in conditioned responding as the presented stimulus differs from CS+. Participants with OC traits are predicted to show linear declines in generalization gradients, suggesting overgeneralization of conditioned fear. In particular, this study is interested in the overestimation of threat since high levels of Threat Estimation as measured by the OBQ-44 are predicted to be associated with the overgeneralization of conditioned fear.

## Methods

### Participants

Participants included 59 adults (38 females, 21 males) whose ages ranged from 18 to 30 years of age. Participants were selected based on their responses to the Obsessive-Compulsive Inventory- Revised (OCI-R), a 18-item questionnaire that measures six dimensions of OCD symptoms including washing, obsessing, hoarding, ordering, checking, and neutralizing [3]. This scale can be used to screen for the frequency of obsessive-compulsive symptoms and to measure symptom severity using a 5-point Likert scale of subjective distress. The OCI-R has been shown to have adequate psychometric properties in both clinical and nonclinical samples [23–25].

A total of 470 undergraduates completed the Obsessive-Compulsive Inventory-Revised [3] using a secure online survey. From these students, 59 individuals met the criteria for this study and were recruited for the psychophysiological recording session based on their total OCI-R score. The present study adopts Foa et al.’s [3] recommendation that a clinically significant cutoff is an OCI-R score of 21 or greater. Using this criterion, two similarly sized groups were selected: a high obsessive-compulsive group (OCI-R ≥ 21) consisting of 28 individuals (16 females, 12 males) and a low obsessive-compulsive group (OCI-R ≤ 20) composed of 31 individuals (22 females, 9 males). Foa Foa et al.’s [3] recommended cutoff score of 21 on the OCI-R does not imply that an individual with a score of 21 or greater would be diagnosed with OCD; instead, a score of 21 or greater suggests that the participant endorses obsessive-compulsive symptoms to a greater extent than expected in a healthy sample. Table 1 shows the demographics for the high and low obsessive-compulsive groups. There were no between-group differences in age.

Overestimation of threat was measured with the Threat Estimation subscale of the Obsessive Beliefs Questionnaire (OBQ-44). The OBQ-44 was developed by the Obsessive Compulsive Cognitions Working Group to measure beliefs that are thought to be related to the maintenance of OCD [26]. It consists of six belief domains: Responsibility, Threat Estimation, Perfectionism, Intolerance of Uncertainty, Importance of Thoughts, and Control of Thoughts. The OBQ-44 has demonstrated convergent validity with measures OCD symptoms and OBQ-44 total scores have been shown to be significantly higher in OCD patients than community controls, student controls, and anxious controls [9]. A factor analysis suggested these items could be grouped into three belief dimensions: Responsibility/Threat Estimation, Perfectionism/ Intolerance of Uncertainty, Importance/Control of Thoughts [9]. The use of the Threat Estimation items as a scale of overestimation of threat is supported by a later factor analysis of the OBQ-44 that suggests that the Threat Estimation subscale is a unique factor [8]. The high Threat Estimation group (Threat Estimation ≥ 21) consisted of 32 individuals (17 females, 15 males) while the low Threat Estimation group (Threat Estimation ≤ 20) consisted of 27 individuals (21 females, 6 males). Table 2 shows the demographics for the high and low Threat Estimation groups. No between-group differences in age were apparent.

All participants had normal or corrected-to-normal vision and hearing and no history of a major neurological condition. Participants were excluded if they were currently using psychoactive medications. This study was approved by the University of Minnesota Institutional Review Board and informed consent was obtained from all participants. Participants received extra credit in an introductory course for their participation.

### Physiological apparatus

Fear-potentiated startle was recorded using a commercial system (PsychLab psychophysiologic recording system, Precision Instruments), which also administered mild electric shocks to the non-dominant wrist of subjects. The shocks were delivered through two-disk electrodes placed on the participants’ non-dominant wrist. Participants received between 1–3 sample shocks prior to the start of the experiment, which they rated in terms of painfulness on a scale from 0 (no pain) to 5 (extremely painful). The shock level was adjusted based on our assessment of their tolerance. The shocks had an intensity between 3 to 5 milliamperes and duration of 100 ms. Fear-potentiated startle (startle blink or electromyography) was measured using two 6mm silver-chloride electrodes positioned under the left eye (sampling rate=1000 Hz; bandwidth=30–500 Hz). A ground electrode was placed on the participant’s non-dominant forearm. To be consistent with previous work using this generalization paradigm [4], startle probes consisted of 40 ms, 102 dBA bursts of white noise with a near instantaneous rise time presented binaurally through headphones.

### Conditioned generalization paradigm

**Figure 1:**
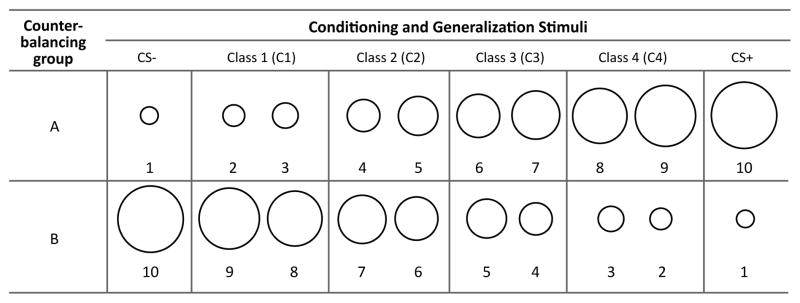
Conditioning and Generalization Stimuli. Groups were counterbalancing so that for half of the participants, the largest ring was the conditioned danger cue and the smallest ring was the safety cue (counterbalancing group A) and for the other half, the stimuli were reversed (counterbalancing group B). The eight intermediate ring sizes were grouped into four classes (C1, C2, C3, and C4) to avoid an excessive number of trials while maintaining a gradual continuum of ring sizes (see Lissek et al., 2010).

The conditioned generalization paradigm used in this study has been described in detail elsewhere [4,16]. This paradigm involves the presentation of 10 rings of gradually increasing size, where the largest and smallest rings serve as the conditioned danger cue (paired with an unconditioned stimulus; CS+) or the conditioned safety cue (not paired with an unconditioned stimulus; CS−). For half of the participants, the largest ring was the conditioned danger cue and the smallest ring was the conditioned safety cue (counterbalancing order A) and for the other half, this was reversed (counterbalancing order B). An electric shock (3–5 mA) delivered to the participant’s non-dominant wrist was used as the unconditioned stimulus. The generalization stimuli consisted of eight intermediately sized rings that form a continuum of similarity between the CS+ and CS− (see Figure 1). Rings were presented for eight seconds on a computer monitor using Presentation software. Prior to the start of the study, participants underwent habituation to nine startle probes.

The conditioned generalization paradigm consists of three phases: preacquisition (presentation of the CS− and CS+ stimuli without shocks), acquisition (fear conditioning with the CS− and CS+), and generalization (presentation of the CS−, CS+, and the eight generalization stimuli). Partial reinforcement of the conditioned danger cue (50% contingency) was used during the generalization phase to avoid extinction of the conditioned response. The trial types and frequencies for each phase are listed in table 3. During each phase, half of the trials were followed by startle probes that occurred 4 or 5 seconds after onset of the conditioned or generalization stimulus. A balanced number of startle probes were presented during inter-trial intervals. Startle probes were separated by 18–25 second time intervals throughout the study, consistent with previous work [4].

During stimulus trials and inter-trial intervals without startle probes, behavioral ratings (perceived risk for shock) and response times were collected. Participants were shown the question “Level of risk?” presented above the stimulus 1 to 2 seconds after trial onset, which cued participants to rate their perceived likelihood of receiving a shock on a 3-point scale (1=no risk, 2=moderate risk, and 3=high risk). Participants were instructed to respond as quickly as possible with their dominant hand using a subject response box. Additionally, retrospective self-reported levels of anxiety evoked by conditioned danger and conditioned safety cues were collected using 10-point Likert scales (1=none, 5=some, 10=a lot) following the acquisition and generalization phases.

### Mood and anxiety questionnaires

Prior to the physiological recording session, participants completed a battery of questionnaires online including the Obsessive Beliefs Questionnaire [9], the Obsessive-Compulsive Inventory-Revised [3], the State Anxiety Inventory [27], and the Beck Depression Inventory [28] as well as a demographics questionnaire.

### Data analysis

Startle electromyography (EMG) raw data was rectified and smoothed using a 20 ms moving window average. The onset latency window for the startle EMG response was 20–100 ms. Peak EMG magnitude was determined by taking the peak value between 21 and 120 ms following stimulus onset (startle probe) and subtracting the average baseline EMG level 50 ms prior to the stimulus onset. For each trial, a zero response was scored if no peak magnitude was detectable (i.e., EMG magnitude less than 1 microvolt). Criteria for trial rejection included unstable baseline EMG activity or peak EMG magnitudes occurring within 20 ms of startle probe onset. The percentage of trials that were rejected based on these criteria was similar in the high and low OCI-R groups as well as the high and low Threat Estimation groups.

EMG magnitudes were standardized using within subjects T-scores. No differences were found between the counterbalancing orders in terms of age, OCI-R scores, Threat Estimation scores, BDI scores, State Anxiety scores, or startle EMG for any of the acquisition or generalization stimuli (*p*-values ≥ 0.15). EMG magnitudes during acquisition phase were analyzed with a 2 × 2 repeated measures analysis of variance (ANOVA): group (high and low OCI-R) by stimulus (danger cue and safety cue). EMG magnitudes during the generalization phase were analyzed with a 2 × 6 repeated measures analysis of variance (ANOVA): group (high and low OCI-R) by stimulus type (safety cue, Class 1, Class 2, Class 3, Class 4, and danger cue). Four individuals were excluded from startle EMG analyses because of equipment problems. ANOVAs were computed using Wilks’ lambda and were followed, when necessary, by either trend analyses or paired-samples t tests. Geisser-Greenhouse corrections were used when there were violations of the sphericity assumption. The shape of generalization gradients were tested using quadratic trend analyses based on a priori predictions that the high OC and high Threat Estimation groups would show a more linear gradient of EMG magnitudes which would reflect greater generalization in these groups. Risk ratings and startle EMG magnitudes were transformed into a measure of deviation from linearity (Mean (CS+ and CS−) − Mean (Classes 1, 2, 3, and 4)) in order to obtain a single continuous measure that characterizes the generalization slope, which can then be correlated with other variables of interest such as symptom questionnaires. Alpha was set at 0.05 and was corrected using Hochberg’s adjustment for multiple tests where appropriate [29]. Effect sizes were estimated using the unbiased estimator *d* [30].

## Results

### Pre-acquisition

During pre-acquisition, no main effects of stimulus type or stimulus type-by-group interactions were found for startle EMG, suggesting that prior to conditioning, there were no group differences in startle reactions for the danger and safety cues in either the high and low OCI-R groups or the high and low Threat Estimation groups (*p*-values ≥ 0.14).

### Acquisition

#### Startle EMG

Means and standard deviations are displayed in table 4 for the high and low OCI-R groups and table 5 the high and low Threat Estimation groups. A 2×2 group-by-stimulus ANOVA revealed significant main effects for stimulus type in the high and low OCI-R groups (*F*(1, 54)=40.77, *p<*0.001) and in the high and low Threat Estimation groups (*F*(1, 54)=39.22, *p<*0.001). Fear potentiated startle was greater for the danger cue than the safety cue in both the high OCI-R (*t*(26)=4.31, *p<*0.001) and low OCI-R (*t*(28)=4.84, *p<*0.001) groups as well as the high Threat Estimation (*t*(30)=4.31, *p<*0.001) and low Threat Estimation (*t*(24)=5.22, *p<*0.001) groups, showing that all groups were able to condition to the danger cue. There were no significant group-by-stimulus interactions during acquisition, suggesting that the strength of the fear potentiated startle did not differ across groups (*p*-values ≥ 0.48).

#### Retrospective anxiety

Conditioning to the danger cue was also apparent using self-reported measures of anxiety administered post-acquisition. Higher levels of anxiety to the conditioned danger cue compared to the conditioned safety cue were reported in both the high OCI-R group (*t*(27)= 7.96, *p<*0.001, danger cue: Mean= 8.04, SD=1.90, safety cue: Mean= 3.68, SD= 2.20) and the low OCI-R group (*t*(30)= 10.83, *p<*0.001, danger cue: Mean= 7.26, SD= 1.95, safety cue: Mean= 2.61, SD= 1.33). Higher levels of anxiety to the conditioned danger cue were also reported in both the high Threat Estimation group (*t*(31)= 9.65, *p<*0.001, danger cue: Mean= 8.06, SD= 1.78, safety cue: Mean= 3.50, SD= 2.11) and the low Threat Estimation group (*t*(26)= 8.86, *p<*0.001, danger cue: Mean= 7.11, SD= 2.04, safety cue: Mean= 2.67, SD= 1.41). No stimulus type-by-group interactions were found (*p*-values ≥ 0.68); however, main effects for group were found (OCI-R: *F*(1, 57)= 7.28, *p=*0.009; Threat Estimation: *F*(1, 57)= 6.74, *p=*0.012) indicating that the high OCI-R group and the high Threat Estimation group reported greater anxiety to both the danger and safety cues than the low OCI-R or low Threat Estimation groups.

### Generalization test

#### Startle EMG

A 2×2 stimulus type-by-group repeated measures ANOVA revealed significant main effects for stimulus type (safety vs. danger cue) in the high and low OCI-R groups (*F*(1, 53)=31.85, *p<*.001) and in the high and low Threat Estimation groups (*F*(1, 53)=33.66, *p<*0.001). Fear potentiated startle was greater for the danger cue than the safety cue in both the high OCI-R (*t*(25)=3.63, *p=*0.001) and low OCI-R (*t*(28)=4.38, *p<*0.001) groups as well as the high Threat Estimation (*t*(29)=3.39, *p*=0.002) and low Threat Estimation (*t*(24)=4.86, *p<*0.001) groups, which suggests that conditioned fear to the danger cue persisted during the generalization test. No significant stimulus type-by-group interactions were found (*p*-values ≥ .29). No gender differences were found in startle EMG magnitudes for any of the stimuli: safety cue, Classes 1–4, or danger cue (*p*-values ≥ 0.40).

**Figure 2:**
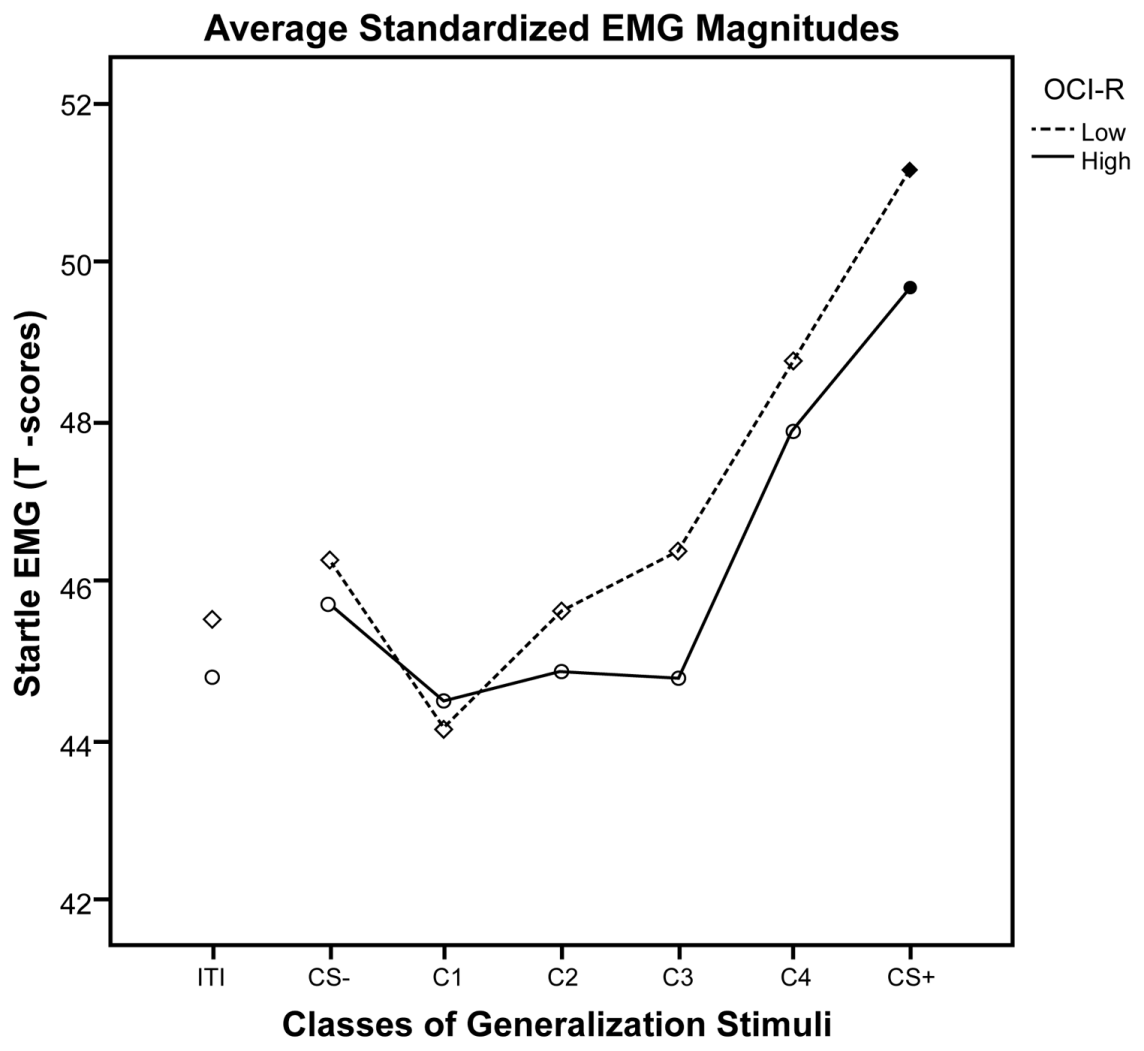
Average standardized startle EMG magnitudes during the generalization test by group (high and low Obsessive-compulsive Inventory-Revised), for the inter-trial interval (ITI), safety cue (CS−), four classes of generalization stimuli (C1, C2, C3, C4), and danger cue (CS+). Black dots indicate that the startle EMG magnitudes were significantly larger for the danger cue than for the safety cue.

**Figure 3:**
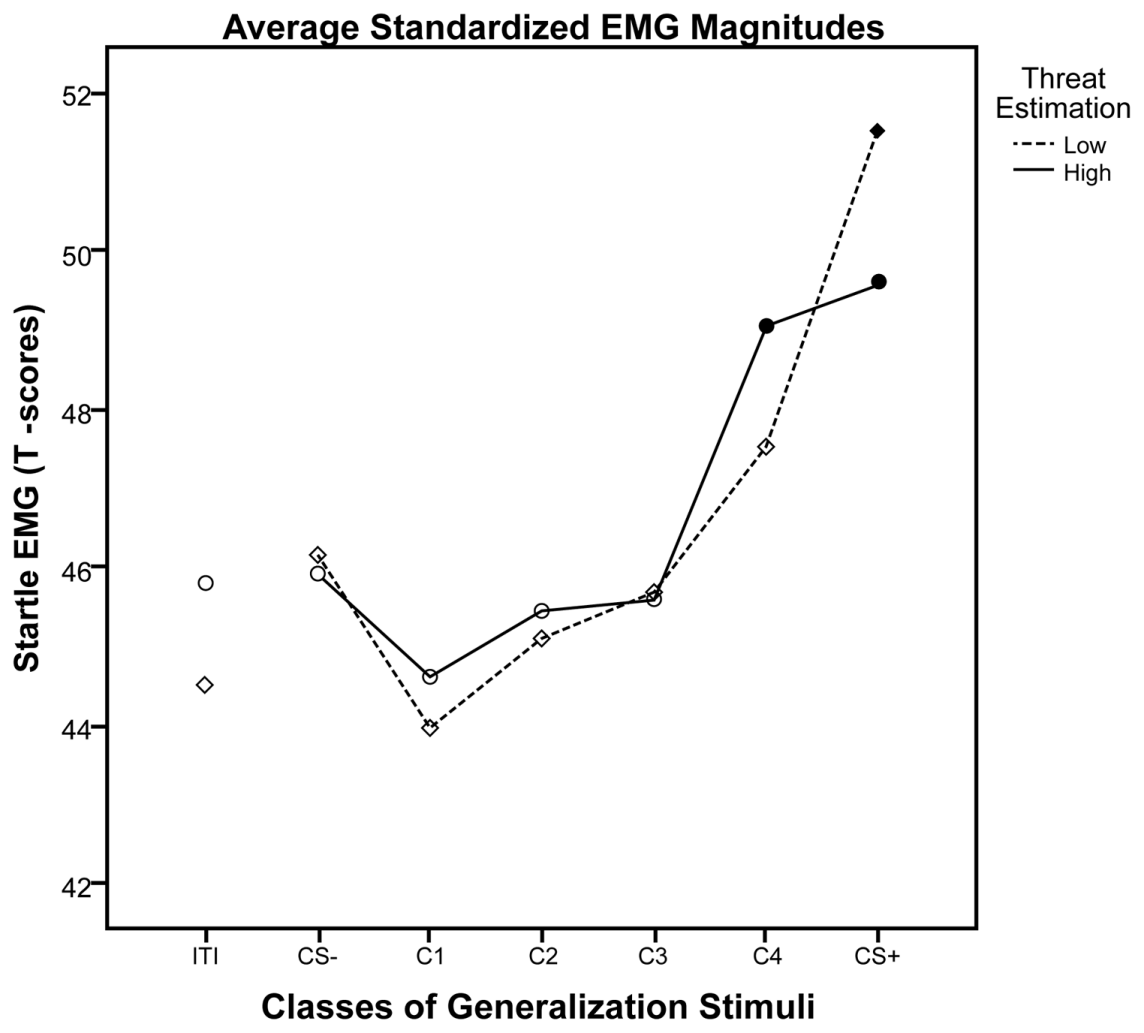
Average standardized startle EMG magnitudes during the generalization test by group (high and low Threat Estimation), for the inter-trial interval (ITI), safety cue (CS−), four classes of generalization stimuli (C1, C2, C3, C4), and danger cue (CS+). Black dots indicate that the startle EMG magnitudes were significantly larger for the danger cue and class 4 (C4) than for the safety cue in the high Threat Estimation group, but were only larger for the danger cue in the low Threat Estimation group.

A 2×6 repeated measures ANOVA revealed main effects of stimulus type for the high and low OCI-R groups (*F*(5,49)=10.44, *p<*0.001) and the high and low Threat Estimation groups (*F*(5,49)=10.91, *p<*0.001). Specifically, the generalization gradients were characterized by downward slopes in startle magnitude as the stimulus becomes less similar to the conditioned danger cue (see Figures 2 and 3). Both the high and low OCI-R groups showed significant quadratic slopes (*F*(1,53)=23.96, *p<*0.001) with no significant stimulus type-by-group interaction (*p*=0.69). The high and low Threat Estimation groups also showed significant quadratic slopes (*F*(1,53)=25.85, *p<*0.001) and there was an interaction between Threat Estimation group and response slopes from the danger cue to stimulus Class 4 (*F*(1,53)=6.30, *p=*0.015). The low Threat Estimation group showed a steep decline in fear potentiated startle between the danger cue and the next class of stimuli (Class 4) while the high Threat Estimation group showed a less steep decline in fear potentiated startle (see Figure 3). As can be seen in Figure 3, this group difference may in part be due to less startle potentiation to the CS+ among high versus low threat estimators, though startle responding to CS+ did not significantly differ by Threat Estimation group, *t*(53)=1.37, *p*=0.18.

Planned comparisons between the conditioned safety cue and the four classes of generalization stimuli, as well as the danger cue, were conducted to determine the point at which startle magnitude was significantly different from the safety cue, indicating that discrimination learning (the opposite of generalization) has occurred. These five contrasts were corrected for multiple comparisons using Hochberg’s adjustment. Using a criterion of *p=*0.02, the results show that startle EMG magnitudes were significantly larger for the danger cue than for the safety cue in both the high OCI-R group (*p=*0.001) and low OCI-R group (*p<*0.001), suggesting that both groups conditioned to the danger cue to the same degree. Similarly, startle EMG magnitudes were also larger for the danger cue than for the safety cue in both the high Threat Estimation group (*p=*0.002) and the low Threat Estimation group (*p<*0.001). Startle EMG magnitudes did not significantly differ between the safety cue and the other classes of generalization stimuli in either the high or low OCI-R groups. In contrast, startle magnitudes were significantly larger for the Class 4 generalization stimuli than for the safety cue in the high Threat Estimation group (*t*(29)=3.14, *p=*0.004) but were not larger relative to the safety cue in the low Threat Estimation group (*t*(24)=1.27, *p=*0.22). The low Threat Estimation group was able to suppress the fear response to the next class of stimuli that resembled the danger cue while the high Threat Estimation group showed similar levels of fear to both the danger cue and the next class of similarly sized stimuli, suggesting overgeneralization of the conditioned fear response.

#### Risk ratings

During generalization, no main effects for group or stimulus type-by-group interactions were found for risk ratings in either the high and low OCI-R groups or the high and low Threat Estimation groups (*p*-values ≥ 0.068). There was a significant main effect for stimulus type in the high and low OCI-R groups and the high and low Threat Estimation groups (*p*-values<0.001). The high and low OCI-R groups and the high and low Threat Estimation groups were characterized by significant quadratic declines in risk ratings as the stimuli decreased in similarity to the conditioned danger cue (*p*-values ≤ 0.005). Risk ratings and startle EMG magnitudes were transformed into a measure of deviation from linearity (Mean (CS+ and CS−) − Mean (Classes 1, 2, 3, and 4)). Using these measures, risk ratings were positively correlated with startle EMG magnitudes (*r*(53)=0.40, *p*=0.003), suggesting that higher risk ratings are associated with more linear (less steep) declines in startle EMG as the stimuli become less similar to the conditioned danger cue.

#### Reaction times

In the high and low OCI-R groups and the high and low Threat Estimation groups, no stimulus type-by-group interactions were found for reaction times (*p*-values ≥ 0.58). No main effect for group was found for the OCI-R groups (*p*=0.37). Although the main effect for group did not reach significance in the Threat Estimation groups either (*p*=0.055), comparisons of the means suggests slower response times in the high Threat Estimation group for all stimuli (safety cue, Classes 1–4, and danger cue). For the reactions times, a significant main effect for stimulus type was found (*p*-values=0.001). All groups were characterized by an inverted U shape, suggesting slower reaction times to the generalization stimuli (Classes 2, 3, and 4), consistent with previous research showing slower responding for stimuli with less certain threat information than the conditioned danger and safety cues [4].

#### Symptom questionnaires

The State Anxiety Inventory was positively correlated with Threat Estimation (*r*(57)=0.52, *p*<0.001) as was the BDI (*r*(56)=.61, *p*<0.001). Correlations between these measures and risk ratings or startle magnitudes were not significant (*p*-values ≥ 0.37).

#### Retrospective anxiety

Self-reported levels of anxiety also show that conditioned anxiety for the danger cue persisted during the generalization test. Higher levels of anxiety to the conditioned danger cue were reported in both the high OCI-R group (*t*(27)=9.35, *p<*0.001, danger cue: Mean=7.86, SD=2.55, safety cue: Mean= 2.32, SD= 1.52) and the low OCI-R group (*t*(30)=15.09, *p<*0.001, danger cue: Mean= 6.77, SD= 2.03, safety cue: Mean= 1.42, SD= 0.67). There was a main effect for group suggesting higher self-reported levels of anxiety to both the danger and safety cues in the high OCI-R group but not the low OCIR group (*F*(1,57)=9.01, *p*=0.004). No stimulus type-by-group interaction was found, suggesting that the pattern of anxiety levels to the danger and safety cues did not differ between the groups (*p*=0.79). Higher levels of anxiety to the conditioned danger cue were also reported in both the high Threat Estimation group (*t*(31)= 11.02, *p<*0.001, danger cue: Mean= 7.63, SD= 2.45, safety cue: Mean= 2.09, SD= 1.47) and the low Threat Estimation group (*t*(26)= 12.30, *p<*0.001, danger cue: Mean= 6.89, SD= 2.17, safety cue: Mean= 1.56, SD= 0.80). No significant main effect for group or stimulus type-by-group interaction was found (*p*-values ≥ 0.07).

## Discussion

This study represents the first attempt to study the generalization of conditioned fear in individuals with obsessive-compulsive traits. The results of this study suggest that individuals with high levels of Threat Estimation as measured by the Obsessive Beliefs Questionnaire (OBQ-44) display overgeneralization of fear responses to a greater range of stimuli resembling the danger cue than those with low levels of Threat Estimation. In particular, the high Threat Estimation group showed greater fear-potentiated startle to ring sizes up to two units of dissimilarity from the danger cue while the low Threat Estimation group did not generalize the conditioned fear response beyond the danger cue. This suggests that the high Threat Estimation group may be characterized by lower thresholds of threat reactivity, which results in greater fear responses to stimuli that resemble the danger cue.

Similar to the animal literature and to previous research on the generalization of conditioned fear in humans [4], this study found that the generalization gradients were characterized by quadratic declines in conditioned fear as the presented stimuli became less similar to the danger cue. Unlike the Lissek et al. [4] study, the present study was not able to replicate the more gradual, linear declines in conditioned fear responding that the authors found in individuals with panic disorder. This divergence in findings may be due to the type of population used in the present study. A limitation to the current study is use of a nonclinical population of individuals with obsessive-compulsive traits rather than patients with OCD. Linear declines in conditioned fear responding may be more apparent when using clinical patients with OCD. It is also possible that linear declines in fear responding are more characteristic of particular anxiety disorders, such as panic disorder and GAD, rather than obsessive-compulsive disorder.

No differences in generalization were found when comparing individuals with high and low levels of overall obsessive-compulsive symptoms as measured by the Obsessive-compulsive Inventory- Revised (OCI-R). Generalization effects in this study were restricted to comparisons between the high and low Threat Estimation groups from the OBQ-44, suggesting that only a subset of individuals with obsessive-compulsive traits show overgeneralization. The lack of group differences in the high and low obsessive-compulsive groups may also be due to the lack of disorder-relevant conditioned stimuli used in this study’s experimental paradigm. Lissek et al. [31] have noted that paradigms that use threat of shock as the unconditioned stimulus may not be as pertinent to anxiety disorders where threat of physical harm is not a key feature. For example, fear of physical harm is characteristic of posttraumatic stress disorder but is less relevant to obsessive-compulsive disorder. Experimental paradigms that use unconditioned stimuli such as contamination may be more successful at finding group differences in fear responses in obsessive-compulsive populations.

Furthermore, the finding of greater generalization in the high Threat Estimation group compared to the low Threat Estimation group and no differences in the high and low obsessive-compulsive groups may be due to overestimation of threat being non-specific to OCD. Research using the OBQ-44 subscales has suggested that beliefs about the importance of thoughts and control of thoughts reliably differentiate OCD patients from other non-obsessive anxiety patients whereas other dimensions such as threat estimation, responsibility, perfectionism, and intolerance of uncertainty may be less specific to OCD [32–35]. In particular, overestimation of threat is common across many anxiety disorders including GAD, OCD, PTSD, and panic disorder. Therefore, we would expect to find overgeneralization in other disorders characterized by high levels of threat reactivity and this has been supported in research on panic and GAD patients and is currently being investigated by the authors in PTSD patients. The current study’s finding of overgeneralization in those high in Threat Estimation coupled with previous research showing greater generalization in anxiety disorders characterized by threat reactivity supports the notion of overestimation of threat possibly being one important process underlying the overgeneralization of fear.

In conclusion, the results of this study suggest that overestimation of threat may be an important precursor to the generalization of conditioned fear. In particular, group differences in conditioned generalization were only found in the high and low Threat Estimation groups as measured by the OBQ-44 and not in the high and low obsessive-compulsive groups as measured by the OCI-R. This suggests that not all individuals with OCD traits are characterized by overgeneralization of fear; specifically, individuals who overestimate threat appear to be at risk for over generalizing their fear. The overgeneralization of conditioned fear remains an important but understudied process in the research on anxiety disorders. Future studies in this area would benefit from the use disorder-specific unconditioned stimuli and from the replication of the current study’s results in a population of OCD patients.

## Figures and Tables

**Table 1:** Group characteristics for the high and low obsessive-compulsive groups

	Low Obsessive-Compulsive	High Obsessive-Compulsive
**Sample size (females, males)**	31 (22, 9)	28 (16, 12)
**Age in years**	20.68 (3.91)	21.29 (3.13)
**OCI-R**	5.63* (6.65)	30.21* (8.59)
**OBQ-44**	132.17* (32.07)	176.36* (33.10)
**BDI**	5.16* (5.72)	17.69* (8.93)
**SAI**	32.84* (9.08)	45.76* (9.12)

*Note.* Group means and (standard deviations) are reported; Significant group differences (p < 0.05) are denoted with an asterisk. OCI-R = the Obsessive-Compulsive Inventory-Revised; OBQ-44 = Obsessive Beliefs Questionnaire; BDI = the Beck Depression Inventory; SAI = State Anxiety Inventory.

**Table 2:** Group characteristics for the high and low Threat Estimation groups

	Low Threat Estimation	High Threat Estimation
**Sample size (females, males)**	27 (21, 6)	32 (17, 15)
**Age in years**	21.19 (4.10)	20.78 (3.06)
**Threat Estimation from the OBQ-44**	14.56* (3.63)	26.88* (5.27)
**OCI-R**	8.83* (9.26)	24.44* (14.38)
**BDI**	6.27* (6.78)	15.11* (9.99)
**SAI**	33.75* (6.61)	43.38* (12.29)

*Note.* Group means and (standard deviations) are reported; Significant group differences (p < 0.05) are denoted with an asterisk. OCI-R = the Obsessive-Compulsive Inventory-Revised; OBQ-44 = Obsessive Beliefs Questionnaire; BDI = the Beck Depression Inventory; SAI = State Anxiety Inventory.

**Table 3:** Trial types and frequencies during preacquisition, acquisition, and generalization test

Conditioning and Generalization Stimuli								
						CS+		
**Phase**	CS−	C1	C2	C3	C4	Coterminated with UCS	Not Coterminated with UCS	ITI
**Preacquisition**	6	-	-	-	-	0	6	6
**Acquisition**	12	-	-	-	-	9	3	12
**Generalization test**	12	12	12	12	12	6	6	12

*Note.* CS−=conditioned safety cue; CS+=conditioned danger cue; C1, C2, C3, and C4=generalization stimulus classes 1, 2, 3, and 4; UCS=unconditioned stimulus; ITI=inter-trial intervals. During the generalization test, the CS+ continued to be reinforced with shock to avoid extinction of the conditioned response during the generalization sequence.

**Table 4:** Acquisition data for standardized startle EMG across conditioned danger cues (CS+), conditioned safety cues (CS−), and inter-trial intervals for the high and low OCI-R groups

	Startle EMGa			
Stimulus	High OCI-R		Low OCI-R	
	Mean	SD	Mean	SD
CS+	54.75	4.51	55.60	4.28
CS−	49.20	4.25	51.16	3.18
ITI	51.22	4.49	49.42	3.06

a Raw startle EMG was standardized with the use of within-subject T score transformations ([([EMG single trial − EMG mean]/SD)*10] + 50). OCI-R = Obsessive-compulsive Inventory-Revised; CS+ = conditioned danger cue; CS− = conditioned safety cue; ITI = inter-trial interval.

**Table 5:** Acquisition data for standardized startle EMG across conditioned danger cues (CS+), conditioned safety cues (CS−), and inter-trial intervals for the high and low Threat Estimation groups as measured by the OBQ-44

	Startle EMGa			
Stimulus	High Threat Estimation		Low Threat Estimation	
	Mean	SD	Mean	SD
**CS+**	55.36	4.60	54.99	4.16
**CS−**	50.07	4.65	50.39	2.55
**ITI**	51.04	4.10	49.36	3.46

a Raw startle EMG was standardized with the use of within-subject T score transformations ([([EMG single trial − EMGmean]/SD)*10] + 50). OCI-R = Obsessive-compulsive Inventory-Revised; CS+ = conditioned danger cue; CS− = conditioned safety cue; ITI = inter-trial interval.
